# Inducible Nitric Oxide Synthase (iNOS): Why a Different Production in COVID-19 Patients of the Two Waves?

**DOI:** 10.3390/v14030534

**Published:** 2022-03-05

**Authors:** Monica Gelzo, Filippo Scialò, Sara Cacciapuoti, Biagio Pinchera, Annunziata De Rosa, Gustavo Cernera, Marika Comegna, Lorella Tripodi, Nicola Schiano Moriello, Mauro Mormile, Gabriella Fabbrocini, Roberto Parrella, Gaetano Corso, Ivan Gentile, Giuseppe Castaldo

**Affiliations:** 1 Ceinge—Biotecnologie Avanzate s.c.a.r.l., 80145 Naples, Italy; gelzo@ceinge.unina.it (M.G.); filippo.scialo@unicampania.it (F.S.); cernera@ceinge.unina.it (G.C.); marika.comegna@unina.it (M.C.); tripodi@ceinge.unina.it (L.T.); gaetano.corso@unifg.it (G.C.); 2 Dipartimento di Medicina Molecolare e Biotecnologie Mediche, Università di Napoli Federico II, 80131 Naples, Italy; 3 Dipartimento di Medicina Traslazionale, Università della Campania L. Vanvitelli, 80131 Naples, Italy; 4 Dipartimento di Medicina Clinica e Chirurgia, Università di Napoli Federico II, 80131 Naples, Italy; sara.cacciapuoti@libero.it (S.C.); biapin89@virgilio.it (B.P.); veghan@gmail.com (N.S.M.); mormile@unina.it (M.M.); gafabbro@unina.it (G.F.); ivan.gentile@unina.it (I.G.); 5 Dipartimento di Malattie Infettive e Emergenze Infettive, Divisione di Malattie Infettive Respiratorie, Ospedale Cotugno, AORN dei Colli, 80131 Naples, Italy; annunziataderosa@yahoo.it (A.D.R.); rob.parrella@gmail.com (R.P.); 6 Dipartimento di Medicina Clinica e Sperimentale, Università di Foggia, 71122 Foggia, Italy

**Keywords:** COVID-19, nitric oxide, steroid therapy

## Abstract

Profound clinical differences between the first and second waves of COVID-19 were observed in Europe. Nitric oxide (NO) may positively impact patients with Severe Acute Respiratory Syndrome CoronaVirus-2 (SARS-CoV-2) infection. It is mainly generated by inducible nitric oxide synthase (iNOS). We studied serum iNOS levels together with serum interleukin (IL)-6 and IL-10 in patients with SARS-CoV-2 infection in the first wave (*n* = 35) and second wave (*n* = 153). In the first wave, serum iNOS, IL-6, IL-10 levels increased significantly, in line with the World Health Organization (WHO) score severity, while in the second wave, iNOS did not change with the severity. The patients of the second wave showed lower levels of iNOS, IL-6, and IL-10, as compared to the corresponding subgroup of the first wave, suggesting a less severe outcome of COVID-19 in these patients. However, in the severe patients of the second wave, iNOS levels were significantly lower in patients treated with steroids or azithromycin before the hospitalization, as compared to the untreated patients. This suggests an impairment of the defense mechanism against the virus and NO-based therapies as a potential therapy in patients with low iNOS levels.

## 1. Introduction

Nowadays, we know that the clinical symptoms of the Coronavirus disease 2019 (COVID-19) infection may present with a heterogeneous clinical phenotype ranging from asymptomatic to mild [1] or severe forms [2] with pulmonary and endothelial inflammation, thromboembolic complications, acute respiratory distress syndrome (ARDS), and multi-organ failure. Although no specific treatments for the disease are available so far, many clinical trials have demonstrated that both antiviral drugs such as remdevisir [3], molnupiravir [4] and paxlovid [5] and monoclonal antibodies treatment [6] can reduce hospitalization, accelerate viral clearance, and improve clinical conditions among hospitalized patients with moderate and severe forms of COVID-19. Nitric oxide (NO) is a reactive oxygen species (ROS) known to inhibit viral replication by cytotoxic reactions that modify viral proteins and nucleic acids [7]. Furthermore, it contributes to the maintenance of normal endothelial function by helping arterial oxygenation and the modulation of inflammatory pathways, preventing the cytokine storm and ARDS. In addition, it has a bronchodilator effect and stimulates mucociliary clearance [8]. For these reasons, NO was used to treat ARDS and other diseases [9], and recently it was used to treat COVID-19 patients [10,11], among which were pregnant women with severe COVID-19 [12].

NO bioavailability is regulated by the activity of three different NO synthases responsible for generating NO from L-arginine. The endothelial NO synthase (eNOS) and the neuronal NO (nNOS) synthase are constitutively expressed, and they play a key role in regulating the vascular tone. In fact, the decreased activity of these enzymes during aging has been shown to be involved in a reduced vascular tone relaxation in elderly [13]. In contrast with eNOS and nNOS, the inducible NOS (iNOS) can produce a large amount of NO and although its increased expression during aging is associated with a deleterious effect [14], it has a pivotal role in fighting infections. In fact, iNOS is expressed by several cells, in particular from all the cells that populate the respiratory tract [15], and the release of NO is induced by cytokines and microorganisms [16] including viruses [8]. Moreover, it has been reported that interleukin (IL)-10 inhibits the induction of iNOS via other cytokines [17].

In Europe, the first wave of COVID-19 pandemic had hit between March and May 2020. Unfortunately, after the lockdown measures during summer 2020, in September 2020 a second wave hit the country with profound clinical differences as compared to the first wave [18,19,20,21]. In this preliminary study, we evaluated the levels of serum iNOS in patients from the two waves together with IL-6, a pro-inflammatory cytokine, and IL-10, relating the results to the severity of the disease and the treatment before recovery.

## 2. Materials and Methods

### 2.1. Patients

Adult patients with a diagnosis of COVID-19 infection and hospital admission during the 1st and the 2nd waves were enrolled. The patients were hospitalized at the Sections of Infectious Diseases of University Hospital Federico II and Cotugno Hospital, Naples. The study was approved by the Ethical Committee of the University Federico II of Naples (protocol code 138/20, April 14, 2020); the impossibility of obtaining an informed consent was the only exclusion criterion in this study. The 35 patients of the 1st wave had a mean ± SD age of 61.2 ± 16.1 years (range: 24–91 years) and included 8 females (23.0%). The 153 patients of the 2nd wave had a mean ± SD age of 48.4 ± 17.3 years (range: 17–86 years) and included 78 females (51.0%). Molecular analysis (RT-PCR) of the nasopharyngeal swab was used to confirm the diagnosis of SARS-CoV-2 infection [22]. The seven ordinal scales made by the World Health Organization (WHO)—Research and Development Blueprint expert group were used to classify the patients considering the worst WHO stage during the infection. In particular, the patients that died during the infection have been classified as WHO 7 [22,23,24].

### 2.2. Methods

All biomarkers were analyzed on serum samples collected from patients on the day of admission to the hospital. Serum IL-6 and IL-10 were determined by Human Magnetic Luminex Assay on Biorad Bio-Plex 100 system (Labospace s.r.l., Milan, 20128, Italy). The limits of sensitivity of IL-6 and IL-10 methods were 1.7 pg/mL and 1.6 pg/mL, respectively. Serum iNOS was analyzed by human NOS2/iNOS ELISA kit (Fine test, Wuhan fine biotech Co., LTD, Wuhan, Hubei, 430206, China) with a limit of sensitivity of 46 pg/mL. All analyses were performed in accordance with the manufacturer’s instructions.

### 2.3. Statistical Analysis

Non-parametric continuous data were reported as median (interquartile range). Shapiro–Wilk test was used to test the normality of distributions. For the statistical analysis of values below the limits of sensitivity, the concentrations were estimated using the following formula of limit of sensitivity/√2 [25]. Comparisons between two groups were performed by Mann–Whitney U test. Multiple comparisons of non-parametric variables were performed by Kruskal–Wallis tests and pairwise differences were evaluated by the Mann–Whitney U test. Linear regression analysis was used to assess the effect of age and gender (independent variables) on IL-6, IL-10 and iNOS (dependent variables) by stepwise method. The frequency and the percentage were used to describe the categorical data. The comparison between groups of categorical variables was performed by using chi-square test.

Receiver operating characteristic (ROC) curve analysis was used and areas under the curve (AUC) were calculated to compare the effectiveness of different molecules to discriminate died patients (WHO 7) from survivor patients (WHO 3–6). According to the criteria of Jones and Athanasiou [26], AUC > 0.97, 0.93–0.96, 0.75–0.92, and 0.6–0.74 were interpreted as “excellent,” “very good,” “good,” and “reasonable,” respectively.

The statistical analysis was performed by SPSS software (version 27, IBM SPSS, Armonk, NY, USA), MetaboAnalyst 5.0 online package [https://www.metaboanalyst.ca, accessed on 15 December 2021] and KaleidaGraph software (v. 4.5.4, Synergy, Reading, PA, USA) was used to obtain graphics. The significance was accepted at the level of *p* < 0.05.

## 3. Results

Table 1 shows the age, gender, and the levels of serum IL-6, IL-10, and iNOS in patients with SARS-CoV-2 infection of the first and the second wave, classified according to the WHO stage. In the first wave we found that 5/35 (14.3%) patients died during the infection (WHO 7), while in the second wave, those who did not survive were 7/153 (4.6%).

The median age and the percentage of males were significantly (*p* < 0.05) lower in the second wave patients of the three WHO subgroups. The levels of serum IL-6, IL-10 and iNOS were significantly (*p* < 0.0004) lower in the patients of the second wave.

Furthermore, in both the waves, the age of the patients was higher in those with a more advanced WHO stage. The percentage of male patients increased significantly along with the increase of the WHO stage. In the first wave, serum IL-6, IL-10, and iNOS levels increased significantly along the WHO stage, while, in the second wave, IL-6 levels were variable, IL-10 showed a decreasing trend and iNOS did not change.

**Figure 1:**
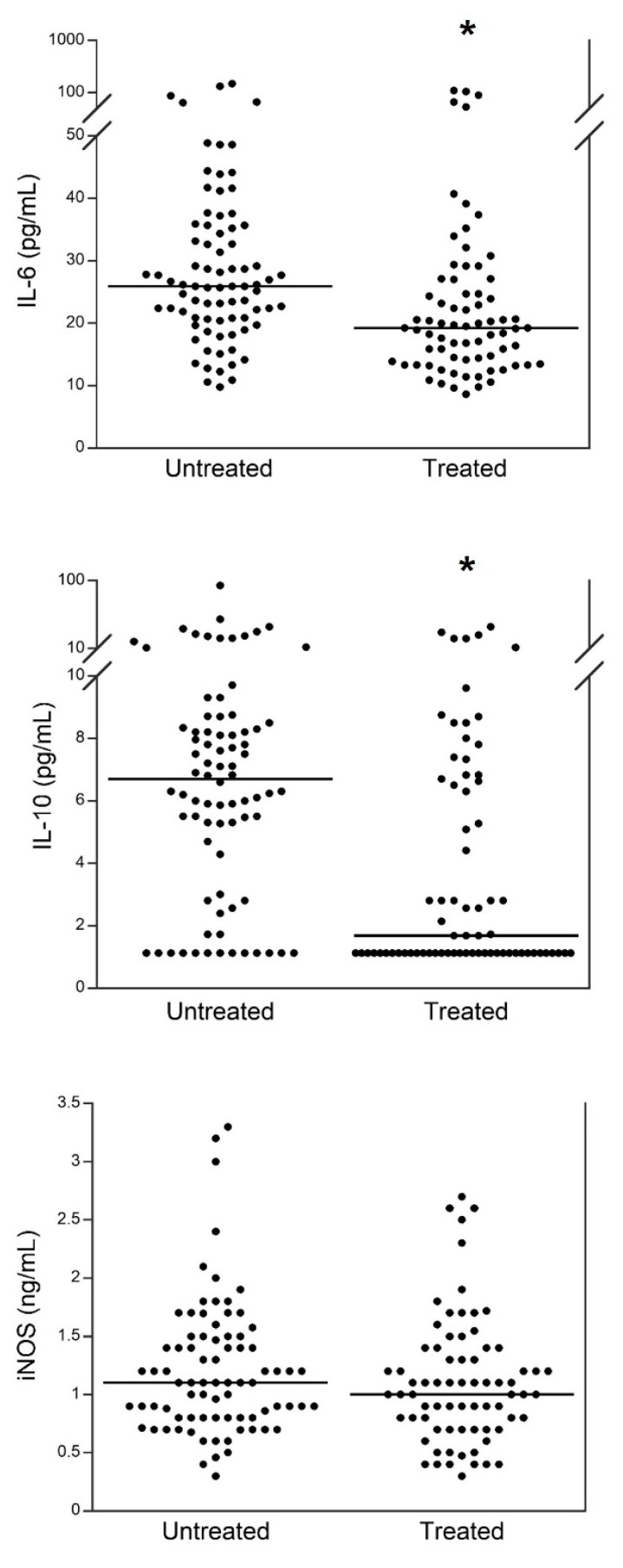
Comparison of serum IL-6, IL-10, and iNOS between 2nd wave patients that have taken steroids and azithromycin before hospital admission and those not treated with either of the two drugs. Black lines correspond to median values. * *p* < 0.0001.

Figure 1 shows the levels of serum IL-6, IL-10, and iNOS in patients of the second wave that had taken steroids and azithromycin before hospital admission compared to those not treated with either of the two drugs. The levels of both serum IL-6 and IL-10 were significantly (*p* < 0.0001) lower in patients treated, while no significant differences were observed for serum iNOS levels.

Table 2 compares the age, gender, and serum levels of IL-6, IL-10, and iNOS between treated and untreated patients of the second wave of different WHO subgroups. The median age gradually increased with the WHO stage both in treated and in untreated patients; furthermore, the age of treated patients was higher in each subgroup. The IL-6 levels were significantly decreased in the treated compared with the untreated patients in the WHO 3 and WHO 4 subgroups, while no differences were observed between treated and untreated patients of the WHO 5–7 subgroup. Furthermore, among treated patients, a clear increasing trend of IL-6 levels was related to the WHO stage, while such a trend was not observed among untreated patients. Serum IL-10 mirrored the trend of IL-6 in the comparison of treated and untreated patients, but no differences were observed among WHO stages in either of the two subgroups (i.e., treated and untreated patients). Finally, for serum iNOS, no significant differences were found between treated and untreated patients of the WHO subgroups 3 and 4. Conversely, treated patients of WHO subgroups 5–7 showed significantly lower levels of iNOS than untreated patients. Furthermore, an increasing trend of serum iNOS, related to the WHO stage, was observed among untreated patients. This trend was not observed in treated patients.

No associations of age and gender vs. the three biomarkers were found either within treated or untreated patients of the 2nd wave (data not shown).

**Figure 2:**
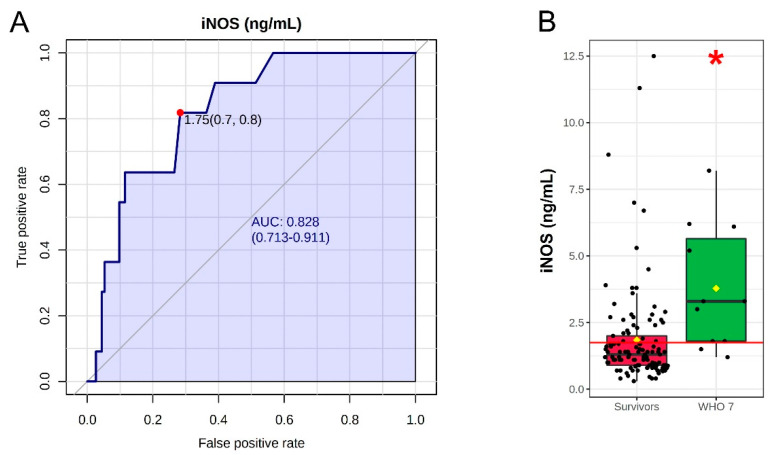
(**A**) Univariate ROC curve analysis for iNOS. (**B**) Box plot of iNOS values in corticosteroid-untreated patients of both waves with WHO 3–6 (survivors, *n* = 113) and WHO 7 (deceased patients, *n* = 11). The red line in box plot represents the best cut-off value. * *p* = 0.0004.

Finally, we performed the ROC analyses for IL-6, IL-10 and iNOS in all patients of the first and second waves that were untreated with corticosteroids. The patients were divided into two groups, survivors (WHO 3–6, *n* = 113) and deceased (WHO 7, *n* = 11). The results showed a poor performance of the ROC curves of both IL-6 and IL-10 with AUC of 0.636 and 0.641, respectively (figures not shown). However, the ROC analysis of the iNOS levels in the same groups showed an AUC of 0.828, with a sensitivity of 82% and specificity of 76% at the best cut-off value of 1.75 ng/mL (Figure 2A). In survivors, the median of iNOS levels (1.3 ng/mL) was about two-fold lower (*p* = 0.0004) than in deceased patients (WHO 7 group; 3.3 ng/mL) (Figure 2B).

## 4. Discussion

We found several differences between patients with SARS-CoV-2 infection of the first and the second wave. In fact, patients from the second wave were significantly younger, although in both the waves, the age of patients was significantly higher in severe stages, confirming that age relates with more severe disease [20]. Furthermore, although we observed a significantly lower percentage of males compared to the first wave, in more severe subgroups of both the waves, the presence of male patients still had a higher percentage, indicating that for some reason the male gender relates with more severe disease [20]. Furthermore, 5/35 (i.e., 14.0%) patients of the first and 7/153 (4.0%) patients of the second wave died during the recovery. All of them were male.

Going to serum biomarkers, we analyzed IL-6, a pro-inflammatory cytokine, and IL-10, an anti-inflammatory cytokine. In addition, it is reported that IL-10 inhibits the induction of nitric oxide synthase via other cytokines [17]. In patients from the first wave, we observed significantly higher levels of serum IL-6, IL-10, and iNOS, as compared to the patients of the second wave, and a significant increase of such values with the progression of the WHO stage [22]. While, among patients of the second wave, the relation between the levels of serum biomarkers and the disease severity was not clear. In particular, we found lower levels of IL-10 in severe/moderate patients of the second wave, as compared to mild patients. This suggests that in the more severe patients of the second wave the anti-inflammatory role of IL-10 is lacking, differently from first-wave patients where a cytokine storm was observed. Other factors may have modulated serum IL-6, IL-10, and iNOS levels in patients of the second wave. For this reason, we explored the impact of the therapies that were prescribed to the patients before hospitalization, and thus before the sampling that was performed in all patients at admission. We observed that the treatment reduced the number of deaths to 1.4% compared to 7.5% of untreated patients in the second wave. All 35 patients of the first wave were diagnosed with SARS-CoV-2 infection by performing RT-PCR analysis on the nasopharyngeal swab (often completed two or three days after the sampling) after the onset of symptoms, and they were hospitalized soon after the result [22]. During the second wave, thanks to the improvement of laboratory tests and the tracing mechanism, most patients could be identified when they were still asymptomatic but had contact with a person infected with SARS-CoV-2. In all cases, the RT-PCR result of the nasopharyngeal test was delivered within one day, and a percentage of patients had taken steroids [27], azithromycin [28] or hydroxychloroquine several days before hospitalization, in some cases with a friendly approach [29]. All steroid-treated patients enrolled in this study started the therapy when they were not yet hospitalized and had a peripheral SpO_2_ < 95% in ambient air. These drugs seem to have an impact on the serum IL levels of our patients. In fact, serum levels of IL-6 and IL-10 in treated patients were lower as compared to untreated ones, according to the well-known inhibitory effect of both the drugs on the cytokine synthesis and release [30,31,32]. However, the treatment was not sufficient to explain the differences in serum IL levels between patients from the two waves. In fact, the serum cytokines in untreated patients of the second wave were significantly lower than those in first wave patients (all untreated). Then, we evaluated the impact of age and gender on the levels of serum biomarkers, since the patients of the second wave were significantly younger and, among them, we recorded a significantly higher number of females. The levels of serum IL-6 and IL-10 did not correlate with the age and with gender of the patients.

Concerning serum iNOS levels, the differences observed between the patients of the first wave (all untreated) and the second wave (48% treated) were partially due to the therapy, since in the second wave (Table 2) we observed a significant difference between treated and untreated patients only for the severe group. However, this finding is in contrast to a previous study reporting that steroids do not influence the expression of iNOS [33]. In addition, serum iNOS levels did not correlate with the age and gender of our patients. Thus, the different levels of serum cytokines and iNOS between patients with SARS-CoV-2 infection from the two waves may also be due to other causes, such as earlier diagnosis and treatment, more rapid hospitalization [18] before the start of the cytokine storm, and before the subsequent increase of serum iNOS or, moreover, the different seasonality of the two waves [21]. Considering all these findings, we performed a biomarker analysis for iNOS in all COVID-19 patients of both waves excluding steroid-treated patients. This analysis showed that iNOS has a good predictive value for COVID-19 outcome [26]. However, the most severely affected, subsequently deceased patients, despite having higher iNOS enzyme levels than the survivors, were unable to control the infection. In these patients, we were unfortunately unable to measure NO levels, and this lack represents a limitation of the study.

The reduction of IL-6 and IL-10, observed in patients of the second wave, confirm that the second wave of SARS-CoV-2 infection was less severe than the first, while the reduction of serum iNOS that we observed in patients with SARS-CoV-2 infection of the second wave, particularly in those with more severe WHO stages, may have impacted negatively on the clearance of the virus. In fact, iNOS activity is the main mechanism to produce NO, which displays a series of beneficial effects in patients carrying acute viral infections [7,8,9]. Such effects seem to occur also in patients with SARS-CoV-2 infection. In fact, the first results among the dozens of ongoing trials on the treatment of patients with SARS-CoV-2 infection with NO [10] reported the percentage of responder patients as high as two-thirds of cases with a significant improvement of respiratory function and reduction of oxygen need [11], and a significant improvement of cardiopulmonary function in six pregnant women with severe COVID-19 treated with high concentrations of inhaled NO [12]. Besides its important function in regulating host defense, the mechanism underlying the benefit of NO use can be related to its key roles in vascular signaling, regulation of blood flow, the prevention of cytokine storms, and restoration of capillary density and oxygen distribution. Therefore, it has been suggested that NO delivery in COVID-19 patients could prevent the progression to a severe condition [34], although more studies are needed to fully comprehend when, how and how much to be administered, in order to obtain its beneficial effects [35].

## 5. Conclusions

Our data confirm the relevant differences between the two waves in terms of age and gender of hospitalized COVID-19 patients. The reduction of serum inflammatory biomarkers in patients of the second wave is a further confirmation of the less severe outcomes of COVID-19 in the second wave, thanks to the improvement of organizational and diagnostic strategies for the SARS-CoV-2 infection. The clinician should pay more attention to severe patients with elevated iNOS levels and, if possible, they should measure the levels of NO. Overall, these results suggest that NO-based therapies should be carefully considered in patients with SARS-CoV-2 infection and with low levels of NO. However, this study is preliminary and further investigations are needed in order to reinforce these conclusions.

## Figures and Tables

**Table 1:** Comparison of serum cytokine levels and iNOS in COVID-19 patients from the 1st wave (*n* = 35) and 2nd wave (*n* = 153) at admission, stratified according to the worst WHO stage. Median and interquartile range.

	Wave	All	WHO 3	WHO 4	WHO 5–7	Multiple Comparison
N	1st	35	7 (20)	20 (57)	8 (23)	-
	2nd	153	57 (37)	58 (38)	38 (25)	-
	1st vs. 2nd	-	0.052	**0.037**	0.806	
Age	1st	62 (50–73)	60 (39–62)	64 (51–73)	75 (58–80)	0.068
(years)	2nd	48 (33–63)	34 (29–43)	53 (38–64) ^a^	56 (48–73) ^b^	**<0.0001**
	1st vs. 2nd	**0.0003**	0.062	**0.019**	0.074	
Males	1st	27 (77)	4 (57)	16 (80)	7 (88)	-
(*n*, %)	2nd	75 (49)	12 (21)	35 (60) ^a^	28 (74) ^b^	-
	1st vs. 2nd	**0.003**	**0.037**	0.111	0.405	
IL-6	1st	171 (94–397)	130 (92–223)	198 (86–375)	292 (53–769) ^b^	**0.021**
(pg/mL)	2nd	22 (16–30)	26 (21–35)	19 (13–25) ^a^	24 (17–36) ^c^	**0.0002**
	1st vs. 2nd	**<0.0001**	**0.0004**	**<0.0001**	**0.0002**	
IL-10	1st	10.1 (5.1–24)	5.4 (4.3–9.1)	13.5 (4.5–24.2)	23.5 (9.7–90.8) ^b^	**0.037**
(pg/mL)	2nd	5.5 (1.13–8.1)	6.5 (5.3–8.2)	2.6 (1.13–7.4) ^a^	2.8 (1.13–8.6)	**0.011**
	1st vs. 2nd	**<0.0001**	0.656	**<0.0001**	**<0.0001**	
iNOS	1st	2.9 (2.3–5.3)	2.3 (1.4–2.6)	2.9 (2.5–4.4) ^a^	6.2 (3.8–7.8) ^b^	**0.007**
(ng/mL)	2nd	1.1 (0.8–1.4)	0.9 (0.7–1.3)	1.2 (0.8–1.5)	1.1 (0.9–1.6)	0.104
	1st vs. 2nd	**<0.0001**	**0.0002**	**<0.0001**	**<0.0001**	

^a^ *p* < 0.01, WHO 4 vs. WHO 3; ^b^ *p* < 0.01, WHO 5–7 vs. WHO 3; ^c^ *p* < 0.01, WHO 5–7 vs. WHO 4. The differences between 1st and 2nd waves were assessed by Mann–Whitney U test. A Chi-square test was used to compare the frequencies. Significant values are reported in bold. “-“: not applied.

**Table 2:** Comparison of serum cytokine levels and iNOS in 153 patients of 2nd wave with different severity according to worst WHO stage and untreated or treated with corticosteroid/azithromycin before hospitalization. Median and interquartile range.

	Wave	WHO 3	WHO 4	WHO 5–7	Multiple Comparison
N	untreated	48	20	12	-
	treated	9	38	26	-
Age	untreated	33 (28–40)	37 (32–61)	49 (41–56) ^a^	**0.012**
(years)	treated	37 (28–63)	57 (48–64)	61 (51–74) ^a^	**0.011**
	*p* value	0.443	**0.003**	**0.043**	
Males	untreated	8 (17)	6 (30)	9 (75) ^a^	-
(*n*, %)	treated	4 (44)	29 (76)	19 (73)	-
	*p* value	0.061	**0.0006**	0.900	
IL-6	untreated	27.3 (22.4–39.8)	22.8 (18.3–28.8) ^b^	22.6 (14.5–36.8) ^a,c^	**<0.0001**
(pg/mL)	treated	17.6 (12.0–20.5)	16.6 (13.0–20.4)	24.3 (18.1–35.7) ^a,c^	**0.001**
	*p* value	**0.0002**	**0.010**	0.582	
IL-10	untreated	6.9 (5.5–8.3)	6.0 (1.9–9.3)	5.4 (1.13–9.9)	0.724
(pg/mL)	treated	1.13 (1.13–6.6)	1.13 (1.13–4.8)	2.8 (1.13–8.1)	0.260
	*p* value	**0.028**	**0.046**	0.540	
iNOS	untreated	0.9 (0.7–1.4)	1.2 (0.8–1.5)	1.6 (1.1–2.0) ^a^	**0.005**
(ng/mL)	treated	0.8 (0.6–1.2)	1.2 (0.8–1.6)	1.0 (0.8–1.1)	0.131
	*p* value	0.258	0.768	**0.005**	

^a^ *p* < 0.01, WHO 5–7 vs. WHO 3; ^b^ *p* < 0.01, WHO 4 vs. WHO 3; ^c^ *p* < 0.01, WHO 5–7 vs. WHO 4. The differences between untreated and treated patients for each WHO subgroup were assessed by Mann–Whitney U test. Chi-square test was used to compare the frequencies. Significant values are reported in bold.

## Data Availability

The data presented in this study are available on request from the corresponding author.
